# Delanzomib, a Novel Proteasome Inhibitor, Combined With Adalimumab Drastically Ameliorates Collagen-Induced Arthritis in Rats by Improving and Prolonging the Anti-TNF-α Effect of Adalimumab

**DOI:** 10.3389/fphar.2021.782385

**Published:** 2021-11-22

**Authors:** Lei Wang, Lixiong Liu, Xiaoping Hong, Dongzhou Liu, Zeneng Cheng

**Affiliations:** ^1^ Department of Rheumatology and Immunology, The Second Clinical Medical College, Jinan University (Shenzhen People’s Hospital), Shenzhen, China; ^2^ Integrated Chinese and Western Medicine Postdoctoral Research Station, Jinan University, Guangzhou, China; ^3^ Research Institute of Drug Metabolism and Pharmacokinetics, School of Xiangya Pharmaceutical Sciences, Central South University, Changsha, China

**Keywords:** adalimumab, collagen-induced arthritis, delanzomib, neonatal Fc receptor, pharmacokinetics

## Abstract

Delanzomib is a novel proteasome inhibitor initially developed for treating multiple myeloma. It was found to inhibit the expression of tumor necrosis factor alpha (TNF-α). This study aimed to investigate the ameliorating effect of delanzomib on collagen-induced arthritis (CIA) and to explore the pharmacodynamics and pharmacokinetics (PK) interactions between delanzomib and adalimumab. Rats with CIA were randomly assigned to receive the treatment with delanzomib, adalimumab, delanzomib combined with adalimumab, or placebo. Visual inspection and biochemical examinations including TNF-α, interleukin 6, and C-reactive protein were performed to assess arthritis severity during the treatment. The adalimumab concentration in rats was determined to evaluate the PK interaction between delanzomib and adalimumab. Also, the levels of neonatal Fc receptor (FcRn) and FcRn mRNA were measured to explore the role of FcRn in the PK interaction between delanzomib and adalimumab. As a result, delanzomib combined with adalimumab exhibited stronger anti-arthritis activity than a single drug because both drugs synergistically reduced TNF-α level *in vivo*. Delanzomib also decreased adalimumab elimination in rats by increasing the level of FcRn. The slower elimination of adalimumab in rats further prolonged the anti-TNF-α effect of adalimumab. Moreover, FcRn level was increased by delanzomib *via* suppressing FcRn degradation rather than promoting FcRn production. In conclusion, delanzomib combined with adalimumab may be a potential therapeutic approach for treating rheumatoid arthritis. The initial finding that the PK interaction occurred between delanzomib and adalimumab may have clinical relevance for patients who simultaneously take proteasome inhibitors and anti-TNF-α therapeutic proteins.

## Introduction

Rheumatoid arthritis (RA) is a complex chronic inflammatory disorder affecting multiple synovial joints (Coras et al., 2020; Wright et al., 2021). The overproduction of proinflammatory cytokines such as tumor necrosis factor alpha (TNF-α), interleukin (IL)-1, and IL-6, has been widely acknowledged as the pivotal factor involved in the pathogenesis of RA (Feldmann et al., 1996; Kanai et al., 2020; Zhang et al., 2021). These cytokines induced by the autoimmune responses against certain self-antigens mediate long-term synovial inflammation and bone destruction (Zhang et al., 2021). They have become important therapeutic targets for the treatment of RA (Maini and Taylor, 2000; Koenders and van den Berg, 2015; Siebert et al., 2015). Many therapeutic proteins have been successfully developed in terms of neutralizing such cytokines. In recent 20 years, TNF-α is one of the predominant targets for the treatment of RA, and many anti-TNF-α drugs, including adalimumab, infliximab, and etanercept, have been successfully developed (Feldmann and Maini, 2001; Bossaller and Rothe, 2013). Further, some new drugs and therapies are being proposed to provide more choices for the treatment of RA (Abbasi et al., 2019).

Proteasome inhibitors were initially developed for treating multiple myeloma by promoting apoptosis in human multiple myeloma cells (Ito, 2020). Recently, they are used for the treatment of some systemic autoimmune diseases (Alexander et al., 2015; Verbrugge et al., 2015; Eskandary et al., 2018; Lassoued et al., 2019; Thibaudeau et al., 2019). Bortezomib, the first approved nonreversible proteasome inhibitor, attenuated murine collagen-induced arthritis (CIA) and was used for the treatment of RA by inhibiting NF-κB activation (Lee et al., 2009; Kohler et al., 2019). As a novel reversible proteasome inhibitor, delanzomib could efficiently induce an improved response against bortezomib-resistant cells, and delanzomib showed an enhanced anti-multiple myeloma activity of bortezomib (Berkers et al., 2012; Sanchez et al., 2012). Delanzomib was found to ameliorate disease symptoms and glomerulonephritis by inhibiting the expression of TNF-α in two preclinical mouse models of systemic lupus erythematosus (Seavey et al., 2012). While working on the same target TNF-α, delanzomib may exert a synergistic effect with other anti-TNF-α therapeutic proteins on the pathophysiology of RA (Xi et al., 2019). Adalimumab is a widely used anti-TNF-α therapeutic protein for the treatment of RA and psoriasis by neutralizing TNF-α or by affecting the expression profiles of genes involved in the pathophysiology of diseases (Wcisło-Dziadecka et al., 2018; Grabarek et al., 2019; Grabarek et al., 2021). Whether delanzomib can be used for the treatment of RA and whether pharmacodynamics interactions exist between delanzomib and adalimumab needs further exploration.

Drug-drug interaction (DDI) usually occurs between chemical drugs (Deng et al., 2020). It significantly affects the efficacy of drugs after changing their pharmacokinetics. Recently, DDI between anti-TNF-α therapeutic proteins and disease-modifying anti-rheumatic drugs has been reported (Ferri et al., 2016; Wang et al., 2018; Deng et al., 2019). The elimination of adalimumab in patients with RA was affected by the concomitant use of methotrexate (Deng et al., 2019). Whether delanzomib affected the pharmacokinetic (PK) of adalimumab when they were co-administered for the treatment of RA needed investigation.

Hence, this study was performed in rats with CIA to assess the effect of the treatment of delanzomib with adalimumab on the arthritis severity of RA. The PK interaction between delanzomib and adalimumab was also evaluated. Further, the possible pathway *via* which delanzomib interacted with adalimumab was explored by clarifying the role of neonatal Fc receptor (FcRn) in the effect of delanzomib on the elimination of adalimumab.

## Materials and Methods

### Materials

Twenty-four rats (Sprague Dawley, Slac Jingda Laboratory Animal Co. Ltd., Changsha, China) (50% female and 50% male, weighing 180–220 g) were kept under a 12 h light-dark cycle at an ambient temperature of 21–22°C, and they were offered standard laboratory diet and water.

The animal studies were approved by the Animal Ethics Committee of the Xiangya Pharmaceutical School of Central South University. All experiments were conducted following the National Institute of Health Guide for the Care and Use of Laboratory Animals.

### Regents

Delanzomib (Aladdin Industrial Corporation, China, Catalog No. D127401-25 MG) and adalimumab (AbbVie, Maidenhead, United Kingdom) were purchased for examining DDIs. The 96-well plates (Greiner, Germany) were obtained for immobilizing recombinant human TNF-α (Peprotech, NJ, United States, Catalog No. 300-01A), which captured adalimumab on the solid-phase surface of plates. The blocking reagent, which consisted of 5% nonfat dried milk (Dingguo Changsheng Biotechnology, China, Catalog No. DH220-2) solution dissolved in phosphate-buffered saline (PBS) containing 0.5% Tween-20 (PBST) (Dingguo Changsheng Biotechnology, China, Catalog No. DH358-3), was used to block the solid-phase surface of plates. The wash solution, comprising 0.5% Tween-20 in PBS, was used to eliminate nonspecific absorbate. Horseradish peroxidase (HRP)-goat anti-human IgG (H + L) conjugate (ABclonal Technology, MA, United States, Catalog No. AS002) was used for detecting the level of adalimumab. Tetramethyl benzidine (TMB) substrate (Solarbio, China, Catalog No. PR1200) was the substrate for HRP, and sulfonic acid (Sinopharm Chemical Reagent, China, Catalog No. 10021618) was diluted to a concentration of 2 M to stop the HRP reaction.

Commercial immunoassay kits (Fangcheng Jiahong Biotechnology, China) were purchased to measure the levels of TNF-α, IL-6, and C-reactive protein (CRP). The FcRn commercial immunoassay kits (Fangcheng Jiahong Biotechnology, China) were used to measure the FcRn level in the tissues of rats. Anti-adalimumab antibody commercial immunoassay kits (Feiya Biotechnology, China) were used for detecting anti-adalimumab antibody. Cycloheximide (Biotopped, China, Catalog No. C2150) was used to suppress protein synthesis. Dulbecco’s modified Eagle’s medium (DMEM, high glucose, Catalog No. 11965092) and fetal bovine serum (FBS, Catalog No 0.10100139C) were purchased from the manufacturer (Gibco, United States). RevertAid first-strand cDNA synthesis kits (K1622, Thermo Fisher, United States) was obtained for the RNA circulation reverse transcription reaction. 2 × Taq SYBRGreen quantitative polymerase chain reaction (qPCR) mix (Innovagene, China, Catalog No. SQ101-01) and real-time PCR primer (Tsingke, China) were purchased for real-time PCR.

### Treatment

#### Induction of Rats With CIA

All rats were intradermally injected with 300 μl of 1 mg/ml bovine type II collagen emulsified in incomplete Freund’s adjuvant (2:1, w/v) into the base of the tail. One week later, the rats were intradermally injected with 150 μl of the aforementioned reagent once again.

#### Treatment for CIA Rats

All rats with CIA were randomly assigned to receive the treatments of delanzomib (DL-treated group), adalimumab (adalimumab-treated group), delanzomib combined with adalimumab (DL + adalimumab-treated group), or placebo (untreated group). The rats in DL-treated group were intravenously injected with 0.1 mg delanzomib once a week, while the rats in the adalimumab-treated group were intraperitoneally injected with 0.25 mg adalimumab once a week. The rats in DL + adalimumab-treated group were simultaneously treated with 0.1 mg delanzomib and 0.25 mg adalimumab. During the same period, the rats in the untreated group were intraperitoneally injected with the same volume of PBS once a week.

#### Assessment of Arthritis Severity

Visual inspection and biochemical examinations were performed to evaluate arthritis severity during the treatment. One paw of the rats was scored from 0 to 4 as follow: 0 for no signs of arthritis; one for erythema and slight swelling in the middle of the foot (tarsal) or ankle joint; two for erythema and slight swelling from the ankle to the middle of the foot; three for erythema and moderate swelling from the ankle to the metatarsal joint; and four for erythema and severe swelling in the ankle, foot, and digits. The score of each rat was defined as the sum of the scores of all four paws. Grading was performed once a week for 8 weeks by two independent observers. The blood of rats was also collected once a week for examining biochemical indicators including TNF-α, IL-6, and CRP.

#### Pharmacokinetics of Adalimumab in the Rats With CIA

After the seventh administration of adalimumab, 1 ml of blood was collected *via* the retro-orbital bleed at 0, 2, 6, 24, 48, 96, 168, 264, and 336 h. The blood samples were kept at 4°C for 30 min, and then the serum samples were centrifuged at 3,000 rpm for 10 min.

The tissues of the liver, kidney, and intestine were harvested after blood samples were collected. After the tissue were weighed, the saline was added at a ratio of 1:3 (w/v) to prepare the tissue homogenate solution. The tissues samples were separated from the supernatant of homogenate at 12,000 rpm for 10 min.

### Assay Methods

#### Enzyme-Linked Immunosorbent Assay for Adalimumab

The concentration of adalimumab in serum samples was detected using enzyme-linked immunosorbent assay (ELISA). The 96-well plates were coated with 1 μg/ml recombinant human TNF-α (100 µl/well) on the solid-phase surface for 12 h at 4°C. The wells were washed three times with PBST solution and blocked with blocking reagent (5% nonfat dried milk in PBST, 250 μl/well) at 37°C for 2 h. Following the removal of the blocking buffer, the serum samples diluted in 1% nonfat dried milk solution were added into the 96-well plate (50 μl/well) to be incubated at 37°C for 1 h. After washing the wells five times with PBST solution, 1 μg/ml HRP goat anti-human IgG (H + L) conjugate (50 μl/well) was incubated at 37°C for 30 min. Subsequently, the TMB substrate was added (100 μl/well) into the wells and incubated for 10 min. Finally, the reaction was stopped with 1 M sulfonic acid (50 μl/well), and the optical density values were read at 450 nm within 15 min. All measurements were performed in duplicates. A calibration curve was prepared from 31.25 to 1,000 pg/ml. The lower limit of quantification of the assay was 31.25 pg/ml. This assay was proved to be accurate and reproducible when adalimumab concentrations were between 31.25 and 1,000 pg/ml with inaccuracy and imprecision below 15%.

#### ELISA for Anti-Adalimumab Antibodies

The commercial ELISA kits were used to detect the level of anti-adalimumab antibodies in the serum samples of rats with CIA. A calibration curve was prepared from 5 to 80 pg/ml. adalimumab did not interfere the determination of anti-adalimumab antibodies.

#### ELISA for FcRn

The concentration of FcRn in the tissue samples of CIA rats was detected with commercial ELISA kits. A calibration curve was prepared from 18.75 to 600 pg/ml.

### Effect of Delanzomib on FcRn Levels

#### Effect of Delanzomib on the Levels of FcRn Gene Expression

The tissue of rats (100 mg) was homogenized in 1 ml of TRIzol reagent at 25°C for 5 min. After centrifugation at 12,000 rpm for 5 min, the supernatants were mixed with 0.2 ml of chloroform and then incubated for 2 min at 25°C. After the mixture was centrifuged at 12,000 rpm for 15 min, the upper layer (500 μl) was transferred to an RNase-/DNase-free microtube and mixed with 0.5 ml of isopropanol. The mixture was incubated at 25°C for 10 min and centrifuged for 10 min. The RNA pellet was precipitated at the bottom of the tube after removing the supernatant from the tube. The pellet was washed with 1 ml of 75% ethanol and centrifuged at 12,000 rpm for 3 min. The pellet was dried with a vent for 3 min after discarding the wash solution. Subsequently, 0.1 ml of RNase-free water was added to dissolve the RNA pellet at the bottom of the tube. Finally, the concentration of dissolved RNA was determined.

The RNA solution in each tube was diluted to the same concentration (90 ng/μl) by adding RNase-free water before cDNA synthesis. The RNA diluent (5.5 μl) was gently mixed with a random hexamer primer (0.5 μl) and incubated at 65°C for 5 min after being centrifuged briefly. Subsequently, 4 μl of mixture containing 2 μl of 5 × Reaction Buffer, 0.5 μl of RiboLock RNase Inhibitor, 1 μl of dNTP Mix (10 mM), and 0.5 μl of RevertAid RT was added and centrifuged briefly. The mixture was incubated for 15 min at 25°C, followed by 60 min at 42°C. Finally, the cDNA was obtained by termination reaction through heating at 70°C for 5 min.

The cDNA solution was diluted to the same concentration (100 ng/μl) by adding RNase-free water for real-time PCR to quantify the mRNA of FcRn. The primer sequence of FcRn mRNA was shown in Table 1. The 20 μl reaction mixture containing 10 μl of 2 × Taq SYBRGreen qPCR Mix, 0.6 μl of FcRn-F (10 μM), 0.6 μl of FcRn-R (10 μM), 6.8 μl of RNase-free water, and 2 μl of cDNA template was added to strip tubes for real-time PCR. The reactions were performed at 95°C for 2 min, followed by 40 cycles at 95°C for 5 s, and finally at 60°C for 30 s. Glyceraldehyde 3-phosphate dehydrogenase (GAPDH) was selected as the housekeeping gene for an endogenous control of the reaction. The 2^−△△Ct^ relative method was used for analyzing changes in expression.

**TABLE 1:** Nucleotide sequence of primers used in the RTqPCR reaction.

mRNA	Nucleotide sequence
*FcRn*	Forward 5′-CTT​CTG​TGC​CTG​TGG​TCG​GAA​TC-3′
	Reverse 5′- GCA​ATA​GGT​CGC​CAG​AGT​CAT​CAC-3′
*GAPDH*	Forward 5′- GGT​GGA​CCT​CAT​GGC​CTA​CA -3′
	Reverse 5′- CTC​TCT​TGC​TCT​CAG​TAT​CCT​TGC​T -3′

#### Effect of Delanzomib on the Degradation of FcRn

Liver hepatocellular carcinoma (HepG2) cells were cultured in six-well plates at a density of 2 × 10^5^ cells/well. They were incubated in DMEM in 10% FBS and in the presence of 5% CO_2_ at 37°C. After being treated with 100 μg/ml cycloheximide for 12 h, HepG2 cells were divided into two parts for receiving different treatments. Delanzomib were dissolved by the solvent (DMSO: normal saline = 1:9, v: v). The cells in the delanzomib-treated group were treated with 2 ml of delanzomib-added media (50 ng/ml) containing 100 μg/ml cycloheximide, while the cells in the control group were treated with 2 ml of blank media also containing 100 μg/ml cycloheximide. Subsequently, the cell samples in each group were collected after the cells were incubated for 4 or 8 h. Each cell sample (in triplicates) was washed twice with PBS (0.01 M, pH = 7.4) and lysed with 200 μl/well cell lysis buffer (PBS: Triton X-100: PMSF = 100:1:1, *v: v: v*) on ice for 30 min with intermittent vortex mixing. Finally, the cell samples were centrifuged at 12,000 rpm for 10 min at 4°C, and the supernatants were separated and frozen at −80°C. The level of FcRn in cells was detected with commercial ELISA kits.

### Data and Statistical Analysis

Statistical comparisons were performed with SPSS 19.0 (IBM, United States) software. The difference in the arthritis score between different groups was evaluated using the Mann-Whitney test. The changes in the levels of TNF-α, IL-6, and CRP between different treatment groups were analyzed using one-way analysis of variance (ANOVA). It was considered statistically different when *p*-value was less than 0.05.

Non-compartmental analysis was performed with WinNonlin 8.1 software (Certara, United States) to calculate the PK parameters of adalimumab including t_1/2_, C_max_, T_max_, AUC_last_, AUC_inf_, V/F, CL/F, and MRT. The mean and standard deviation of each parameter were calculated for statistical analysis. The difference in T_max_ between the delanzomib-treated and control groups was analyzed using the Mann-Whitney test. The differences in the level of FcRn and in the PK parameters of adalimumab, including t_1/2_, C_max_, V/F, and CL/F between two cohorts were calculated using ANOVA. The least significance difference method was used in the post hoc test. It was considered statistically different when *p*-value was less than 0.05.

## Results

### Induction of Rats With CIA

**FIGURE 1:**
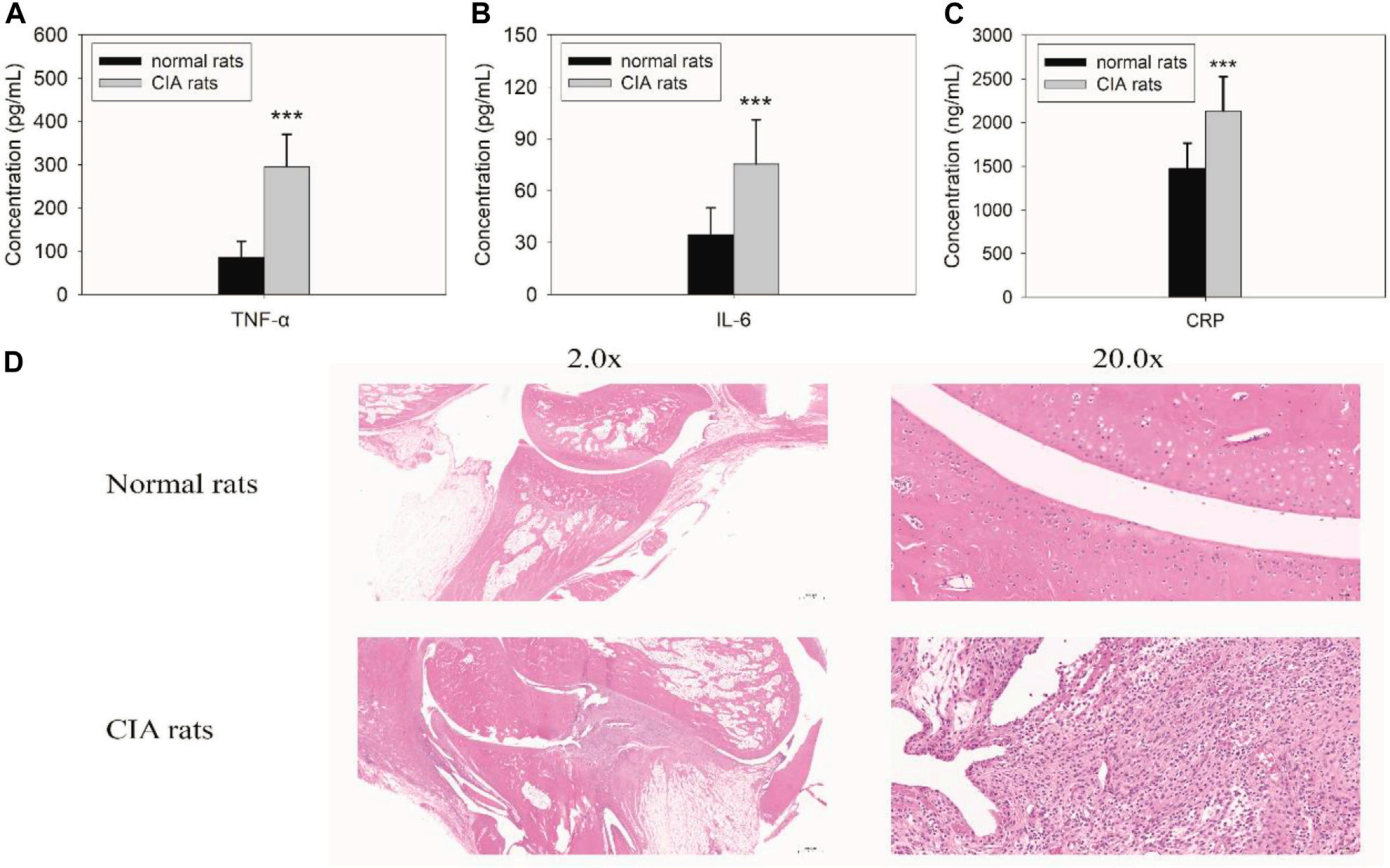
Biochemical examinations for tumor necrosis factor alpha (TNF-a) **(A)**, IL-6 **(B)**, and C-reactive protein (CRP) **(C)** and histopathological findings **(D)** in rats with collagen-induced arthritis (CIA). The levels of TNF-α (*F* = 157.594, *p* = 0.000), IL-6 (*F* = 48.104, *p* = 0.000), and CRP (*F* = 18.426, *p* = 0.000) significantly increased in rats with CIA. Pathological changes in arthritis were also observed during the histopathologic examination of the joints in rats with CIA.

The rats were injected with bovine type II collagen emulsified in complete Freund’s adjuvant for 2 weeks. The levels of inflammatory cytokines, including TNF-α and IL-6, *in vivo* were remarkably increased (Figure 1). The level of TNF-α significantly increased from 85.7 ± 37.5 pg/ml to 295.7 ± 72.9 pg/ml (*F* = 157.594, *p* = 0.000), and the level of IL-6 increased from 34.5 ± 14.9 pg/ml to 75.4 ± 24.8 pg/ml (*F* = 48.104, *p* = 0.000). The level of another inflammatory biomarker CRP also changed accordingly. It increased from 1,491.0 ± 259.6 ng/ml to 2083.3 ± 313.6 ng/ml (*F* = 18.426, *p* = 0.000). Similarly, pathological changes in arthritis were obvious as displayed from the histopathologic examination of the joints in rats (Figure 1D). All the results proved that the induction of rats with CIA was successfully achieved.

### Delanzomib Combined With Adalimumab Decreased the Severity of Arthritis in Rats With CIA

**FIGURE 2:**
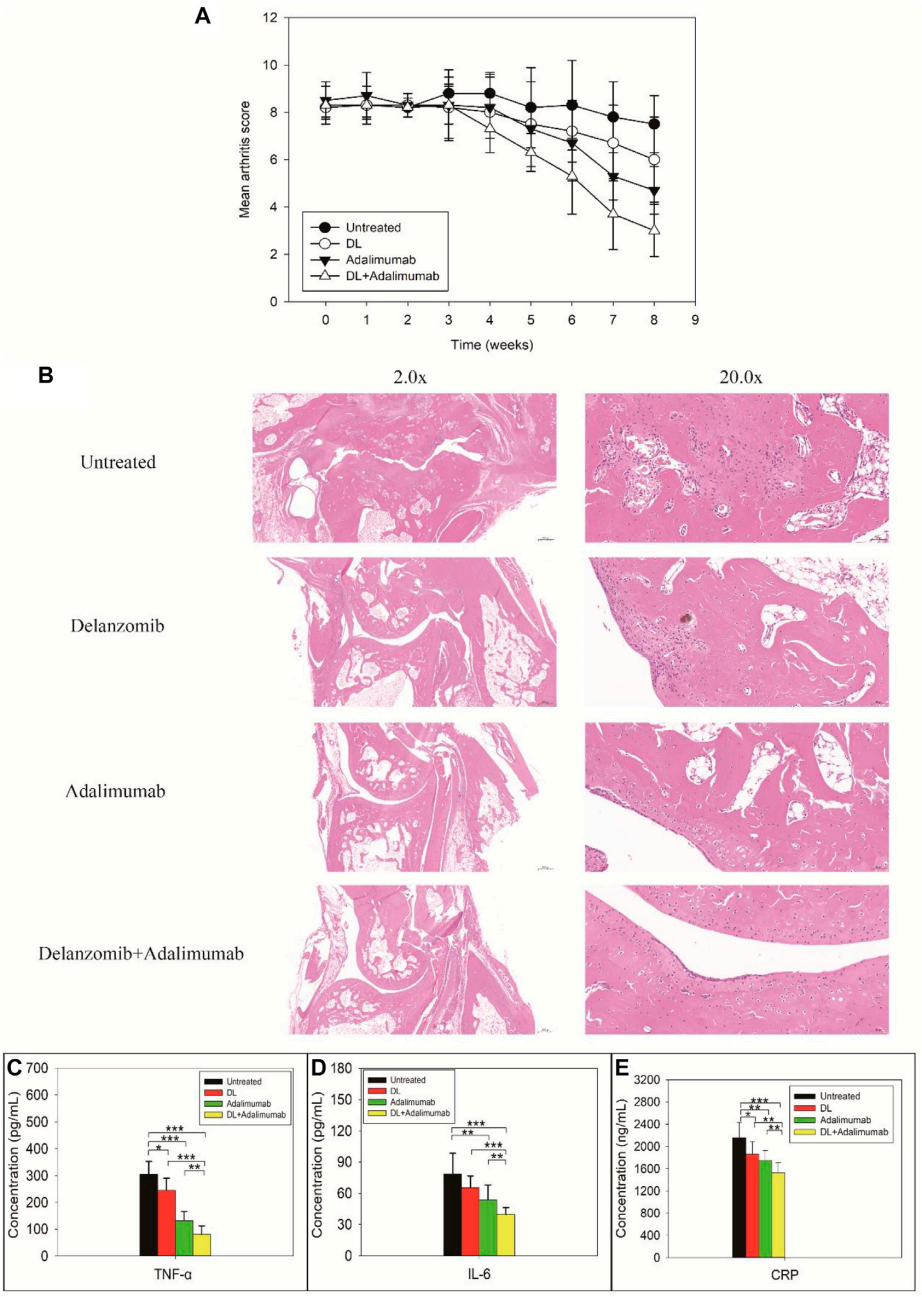
Severity of arthritis **(A)**, histopathological findings **(B)**, and biochemical examinations for tumor necrosis factor alpha (TNF-a) **(C)**, IL-6 **(D)**, and C-reactive proteins (CRP) **(E)** in rats with collagen-induced arthritis after the rats received different treatments. Stronger anti-arthritis activity was observed in the delanzomib + adalimumab (DL + adalimumab)-treated group than in the adalimumab-treated group with lower arthritis score obtained in week eight following the drug administration (*Z* = -2.166, *p* = 0.030) **(A)**. Limited inflammatory infiltration and little or no bone erosion were observed in the joints of DL + adalimumab-treated rats **(B)**. The levels of TNF-α (C), IL-6 (D), and CRP **(E)** significantly decreased in the DL + adalimumab-treated rats than in the rats treated with a single drug.

The arthritis score in DL-treated group, adalimumab-treated group, and DL + adalimumab-treated group began to decrease in week four following the treatment (lasted for 8 weeks for each treatment) (Figure 2A). Especially, the arthritis score in adalimumab-treated (*Z* = −2.677, *p* = 0.007) and DL + adalimumab-treated groups (*Z* = −2.950, *p* = 0.003) remarkably decreased compared with that in the untreated group; however, such a change was not obvious in the DL-treated group (*Z* = −1.526, *p* = 0.127). Importantly, stronger anti-arthritis activity was observed in the DL + adalimumab-treated group compared with the adalimumab-treated group with a lower arthritis score obtained in week eight following drug administrations (*Z* = −2.166, *p* = 0.030).

The results from the histopathologic examinations of the joints were consistent with those from the evaluation of arthritis in the animals. Massive inflammatory infiltration, extensive synovial thickening, and evident bone erosion were observed in the joints of the untreated rats (Figure 2C). In comparison with the control, limited inflammatory infiltration and little or no bone erosion were observed in the joints of DL + adalimumab-treated rats.

### Delanzomib Combined With Adalimumab Reduced the Levels of TNF-α, IL-6, and CRP *in Vivo*

The level of TNF-α in rats with CIA remarkably reduced after treatments with delanzomib (*F* = 4.814, *p* = 0.053), adalimumab (*F* = 50.721, *p* = 0.000), or delanzomib combined with adalimumab (*F* = 89.960, *p* = 0.000). Especially, the level of TNF-α was much lower in the DL + adalimumab-treated group than in the delanzomib-treated group (*F* = 53.173, *p* = 0.000) and adalimumab-treated group (*F* = 7.401, *p* = 0.022), indicating that stronger anti-TNF-α activity was achieved by the concomitant use of delanzomib with adalimumab. The level of IL-6 in rats with CIA also decreased after the rats received different treatments (adalimumab, *F* = 6.317, *p* = 0.031; delanzomib combined with adalimumab, *F* = 21.037, *p* = 0.001). The level of IL-6 was lower in the DL + adalimumab-treated group than in the delanzomib-treated group (*F* = 21.304, *p* = 0.001) and adalimumab-treated group (*F* = 5.095, *p* = 0.048). Similarly, the level of CRP significantly dropped after the CIA rats were treated with delanzomib (*F* = 4.142, *p* = 0.069), adalimumab (*F* = 9.249, *p* = 0.012), or delanzomib combined with adalimumab (*F* = 23.237, *p* = 0.001). The level of CRP reduced more in the DL + adalimumab-treated group than in the delanzomib-treated group (*F* = 8.554, *p* = 0.015) and adalimumab-treated group (*F* = 5.257, *p* = 0.045). The results of aforementioned biochemical examinations suggested that the treatment of delanzomib and adalimumab exhibited stronger anti-arthritis activity than treatment with a single drug.

### Delanzomib Affected the Pharmacokinetics of Adalimumab

The PK parameters of adalimumab in the adalimumab-treated and DL + adalimumab-treated groups are listed in Table 2. The T_max_ of adalimumab changed little after the co-treatment with delanzomib (*Z* = −0.527, *p* = 0.598), indicating that delanzomib affected little on the absorption of adalimumab in rats. Similarly, the parameter of V/F in the DL + adalimumab-treated group was close to that in the adalimumab-treated group (*F* = 0.538, *p* = 0.480). This result suggested that the distribution of adalimumab was not affected by delanzomib.

**TABLE 2:** PK parameters of adalimumab.

Parameters	Adalimumab treated group	Delanzomib with adalimumab treated group
t_1/2_ (h)	82.3 ± 14.1	169.8 ± 83.3
T_max_ (h)	52.0 ± 23.6	44.0 ± 9.8
C_max_ (μg/ml)	9.6 ± 1.5	12.5 ± 1.6
AUC_0-τ_ (h*μg/mL)	1,234.3 ± 205.9	1,629.4 ± 292.8
V/F (ml)	17.0 ± 3.4	18.1 ± 1.7
CL/F (ml/h)	0.21 ± 0.03	0.16 ± 0.02

However, the CL/F of adalimumab was markedly higher in the adalimumab-treated group than in the DL + adalimumab-treated group (*F* = 8.414, *p* = 0.016). In addition, the parameter of t_1/2_ was significantly lower in the adalimumab-treated group (82.3 ± 14.1 h) than in the DL + adalimumab-treated group (169.8 ± 83.3 h). The aforementioned results indicated that the elimination of adalimumab in rats was slowed by the co-treatment of delanzomib. Moreover, the C_max_ of adalimumab was much higher in the DL + adalimumab-treated group than in the adalimumab-treated group (*F* = 10.878, *p* = 0.008). This result implied that the level of adalimumab in the DL + adalimumab-treated group might beyond the lowest effective concentration for a longer duration compared with that in the adalimumab-treated group, especially when the elimination of adalimumab was also remarkable slowed by delanzomib. The alteration in the pharmacokinetics of adalimumab suggested that delanzomib might prolong the anti-TNF-α effect of adalimumab.

### Delanzomib did Not Inhibit the Production of Anti-Adalimumab Antibodies

The concentration of anti-adalimumab antibodies was significantly elevated in the adalimumab-treated (*F* = 5.529, *p* = 0.041) and DL + adalimumab-treated groups (*F* = 7.412, *p* = 0.021) compared with the control group, indicating that the treatment of adalimumab triggered an immune response to produce anti-adalimumab antibodies. However, the concentration of anti-adalimumab antibodies in the DL + adalimumab-treated group was approximately equal to that in the adalimumab-treated group (*F* = 0.029, *p* = 0.868). This result suggested that delanzomib might not inhibit the production of anti-adalimumab antibodies.

### Delanzomib Increased the Level of FcRn

**FIGURE 3:**
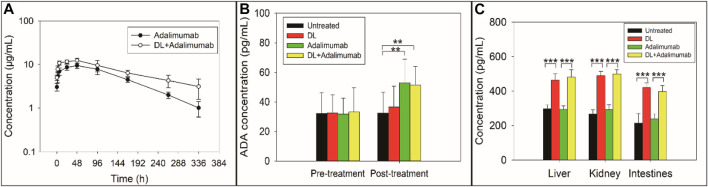
Concentration-time curve of adalimumab **(A)**, concentration of anti-adalimumab antibody (ADA) **(B)**, and concentration of the neonatal Fc receptor (FcRn) **(C)** in rats with collagen-induced arthritis after the rats received different treatments. The elimination of adalimumab in rats was slowed by the co-treatment with delanzomib **(A)**. Delanzomib less affected the production of ADA **(B)**. The ADA level in the adalimumab-treated group was approximately equal to that in the DL + adalimumab-treated group (*F* = 0.029, *p* = 0.868). The level of FcRn in rats was elevated by the treatment of delanzomib **(C)**. The level of FcRn was remarkable higher in the delanzomib-treated group than in the control group (liver, *F* = 107.259, *p* = 0.000; kidney, *F* = 274.189, *p* = 0.000; intestine, *F* = 83.496, *p* = 0.000). Similarly, the level of FcRn was also higher in the DL + adalimumab-treated group than in the adalimumab-treated group (liver, *F* = 119.257, *p* = 0.000; kidney, *F* = 214.382, *p* = 0.000; intestine, *F* = 92.354, *p* = 0.000).

The levels of FcRn in the tissues of liver, kidney, and intestine were remarkable higher in the delanzomib-treated group than in the control group (liver, *F* = 107.259, *p* = 0.000; kidney, *F* = 274.189, *p* = 0.000; intestine, *F* = 83.496, *p* = 0.000) (Figure 3C). Similarly, the level of FcRn was also higher in the DL + adalimumab-treated group than in the adalimumab-treated group (liver, *F* = 119.257, *p* = 0.000; kidney, *F* = 214.382, *p* = 0.000; intestine, *F* = 92.354, *p* = 0.000). However, no considerable difference was found in the level of FcRn between the adalimumab-treated and control groups (liver, *F* = 0.098, *p* = 0.761; kidney, *F* = 3.137, *p* = 0.107; intestine, *F* = 1.238, *p* = 0.292). These results indicated that the level of FcRn in rats was elevated by the treatment of delanzomib.

### Delanzomib Suppressed the Degradation of FcRn but Less Affected the Production of FcRn

**FIGURE 4:**
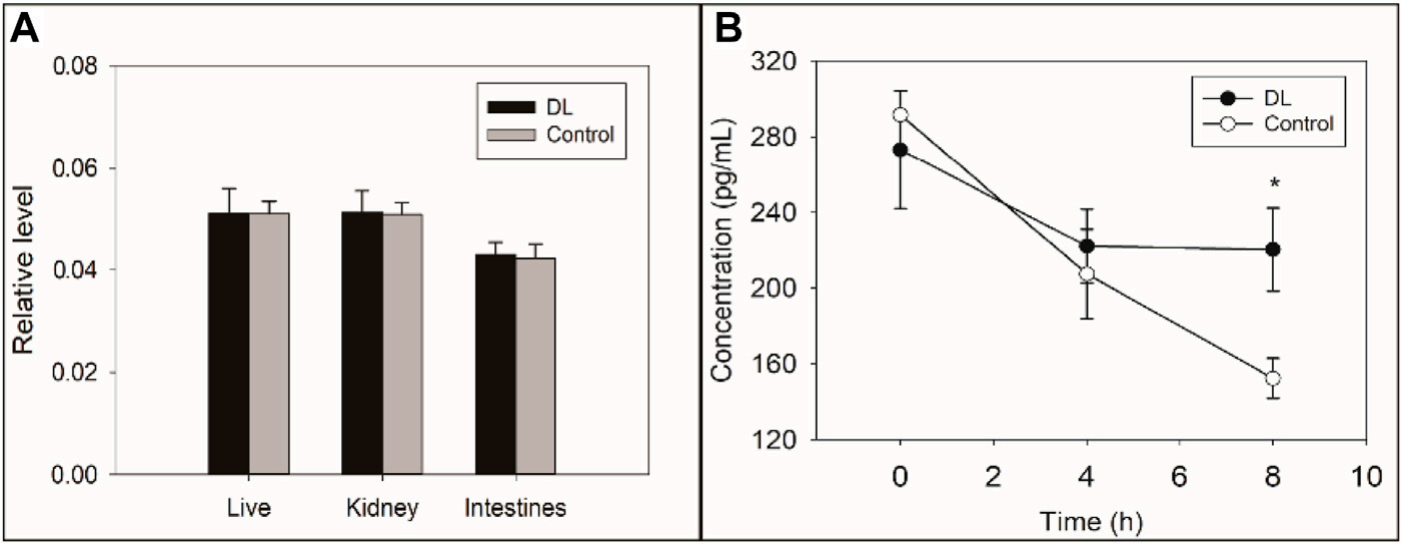
Effects of delanzomib on the production **(A)** and degradation **(B)** of the neonatal Fc receptor (FcRn). Delanzomib did not promote the production of FcRn because the relative level of FcRn mRNA did not increase much after the treatment of delanzomib (liver, *F* = 0.000, *p* = 0.992; kidney, *F* = 0.021, *p* = 0.892; intestine, *F* = 0.152, *p* = 0.717) **(A)**. On the contrary, the degradation of FcRn was suppressed by delanzomib **(B)**. After the cells were treated with delanzomib for 8 h, the level of FcRn was considerably higher compared with that in the control group (*F* = 23.470, *p* = 0.008).

The relative level of FcRn mRNA was not significantly changed after the treatment of delanzomib (liver, *F* = 0.000, *p* = 0.992; kidney, *F* = 0.021, *p* = 0.892; intestine, *F* = 0.152, *p* = 0.717) (Figure 4A), suggesting that delanzomib might not promote the production of FcRn. On the contrary, the level of FcRn in the HepG2 cells of the control group remarkably decreased from 4 to 8 h (*F* = 13.576, *p* = 0.021), but this change was not remarkable in the cells of the delanzomib-treated group over the same period (*F* = 0.012, *p* = 0.918) (Figure 4B). Accordingly, the level of FcRn was considerably higher in the delanzomib-treated group than in the control group after 8 h (*F* = 23.470, *p* = 0.008). These results suggested that delanzomib suppressed the degradation of FcRn in HepG2 cells.

## Discussion

The overproduction of proinflammatory cytokines, such as TNF-α, IL-1, and IL-6, is pivotal in the pathophysiology of RA (Mcinnes and Schett, 2011). CIA is one of the most used models for the stimulation of the pathogenesis of RA (Brand et al., 2007). In this model, antibodies against type 2 collagen (IIC) play a crucial role in the development of arthritis. After IIC was injected into the rats, the autoimmune responses against IIC were triggered to over-produce proinflammatory cytokines, including TNF-α, IL-6, and so on, which realistically simulated the pathogenesis of RA in humans (Brand et al., 2007; Miyoshi and Liu, 2018). In this study, rats with CIA were successfully developed and subsequently, the levels of TNF-α and IL-6 in rats significantly improved and pathological changes on arthritis obviously displayed.

Different treatments were tried to ameliorate the severity of RA. The treatment with delanzomib and adalimumab drastically attenuated the severity of arthritis in rats with CIA. The mean arthritis score and the levels of inflammatory cytokines, including TNF-α, were significantly lower in rats treated with delanzomib and adalimumab than in those treated with either delanzomib or adalimumab. CIA in rats shares various immunological and pathological characteristics with human RA. Hence, the strong anti-arthritic effect of the treatment with delanzomib combined with adalimumab in rats with CIA might suggest the therapeutic potential of the combination for treating RA. Besides, the alleviation of the severity of arthritis in rats with CIA was in accordance with the reduction of levels of inflammatory cytokines. It implied that those cytokines might be potential molecular markers for the evaluation of the efficacy of therapies for treating RA.

**FIGURE 5:**
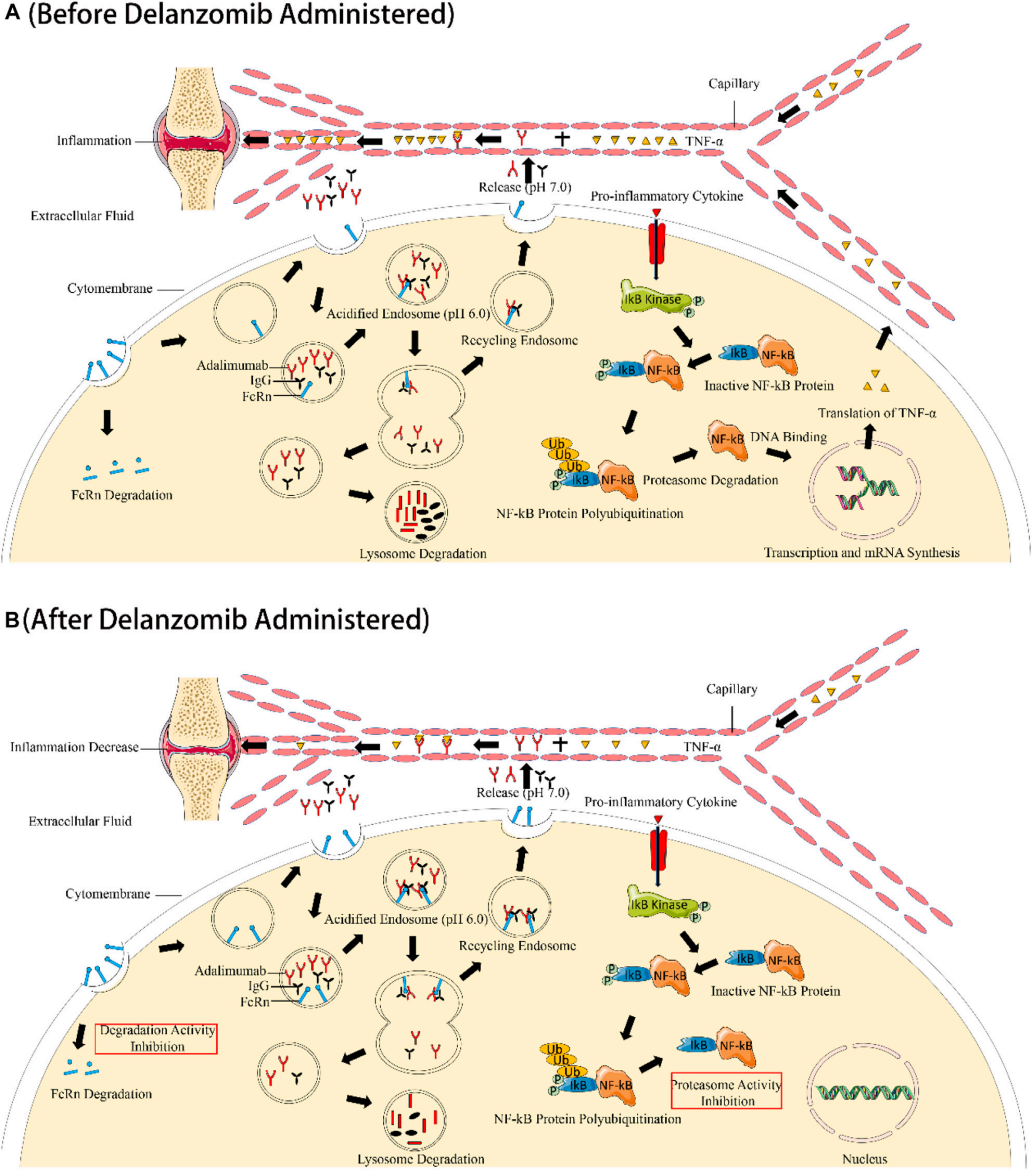
Illustration of the synergistic effect of delanzomib and adalimumab to improve the anti-TNF-α effect. Without the co-administration of delanzomib, the salvaging function of FcRn was normal to allow a certain amount of adalimumab retention in the systemic circulation. TNF-α was also activated by NF-κB under the stimulation of proinflammatory cytokines **(A)**. However, TNF-α was drastically inhibited when adalimumab was combined with delanzomib. On the one hand, delanzomib inhibited NF-κB activation by blocking the ubiquitin-mediated degradation of I-κB, increasing the number of NF-κB and I-κB complexes that cannot enter the nucleus. Delanzomib finally decreases the expression of TNF-α by reducing NF-κB DNA-binding activity. On the other hand, delanzomib slowed down the degradation of FcRn by inhibiting the activity of the proteasome. More adalimumab could be protected from the degradation of the lysosome by FcRn. Eventually, TNF-α was further eliminated by adalimumab through triggering antibody-dependent cellular cytotoxicity or complement activation **(B)**.

The alleviation of CIA in rats by co-administration of delanzomib and adalimumab was probably induced by the synergistic inhibition of TNF-α by both drugs (Figure 5). On the one hand, delanzomib is a novel proteasome inhibitor that inhibits NF-κB activation by blocking the ubiquitin-mediated degradation of I-κB by the proteasome, increasing the number of NF-κB and I-κB complexes that cannot enter the nucleus (Piva et al., 2008; Berkers et al., 2012). TNF-α has been proved to be activated by NF-κB (Beg and Baltimore, 1996). It is thus reasonably concluded that delanzomib decreases the expression of TNF-α by reducing NF-κB DNA-binding activity. For this reason, delanzomib may be available for the treatment of some systemic autoimmune diseases including RA, psoriasis vulgaris, atopic dermatitis, and so on, where TNF-α plays an important role in the pathophysiology. On the other hand, adalimumab is a widely used monoclonal antibody that targets against the proinflammatory cytokine TNF-α or affects the expression profiles of genes involved in the pathophysiology of diseases for the treatment of inflammatory and autoimmune diseases including RA and psoriasis (Bang and Keating, 2004; Fleischmann et al., 2019; Grabarek et al., 2019; Grabarek et al., 2021). TNF-α was eliminated by binding to adalimumab to trigger antibody-dependent cellular cytotoxicity or complement activation (Bang and Keating, 2004). For the aforementioned reasons, the TNF-α level in rats was significantly decreased by an inhibition of the expression of TNF-α through inhibiting NF-κB activation by delanzomib, as well as a promotion of the elimination of TNF-α through binding TNF-α with adalimumab. The interesting finding of this study suggested that the treatment of delanzomib combined with adalimumab might have a reasonable therapeutic effect on RA by synergistically reducing the level of TNF-α *in vivo*.

This was the first report of the pharmacokinetic interaction between delanzomib and adalimumab, likely mediated by the effect of delanzomib on the levels of FcRn in tissues. The elimination of adalimumab probably involved two pathways (Deng et al., 2019). One of the elimination pathways comprised drug endocytosis, endosome trafficking, and lysosome metabolism, similar to the pathway undergone by most xenobiotics. The other one was the elimination mediated by binding to target antigen TNF-α, which could trigger antibody-dependent cellular cytotoxicity or complement activation. FcRn played a key role in protecting monoclonal antibodies (mAbs) from lysosomal metabolism due to the pH-dependent affinity of FcRn to mAbs (Martins et al., 2016; Mackness et al., 2019). MAbs bound to FcRn in the acidified endosome (pH = 6.0), and then mAbs separated from FcRn to be recycled into the circulation after the endosome moved to the cellular surface (pH = 7.0). Other mAbs unbound with FcRn were degraded in the lysosome after endocytosis. The salvaging function of FcRn could extend the half-life of adalimumab (Mackness et al., 2019). Besides, IgG is also protected from proteolysis by binding with FcRn after uptake into endothelial cells. The elimination of IgG autoantibodies targeting structural proteins of the skin will decrease when more FcRn bind with IgG autoantibodies. For this reason, the over expression of FcRn may exacerbate some autoantibody mediated diseases by increasing circulating autoantibody concentrations (Kasprick et al., 2020).

Interestingly, the levels of FcRn in the tissues of the liver, kidney, and intestine increased under the effect of delanzomib, which allowed the recycling of more adalimumab to the systemic circulation by binding with FcRn. Accordingly, lower CL/F value and longer half-life of adalimumab were observed when adalimumab was co-administered with delanzomib. The lower CL/F value and the longer half-life of adalimumab in rats with CIA helped the retention of adalimumab for a longer duration to exert the continuous effect of eliminating TNF-α. Hence, the pharmacokinetic interaction between delanzomib and adalimumab might further prolong the anti-TNF-α effect of adalimumab through slowing the elimination of adalimumab under the action of delanzomib.

Anti-drug antibodies (ADAs) are considered as an important factor for the elimination of mAbs. MAbs, even humanized and fully human mAbs, can act as xenobiotics to activate the formation of endogenous ADAs. The formation of mAb-ADA immune complexes can accelerate the clearance of mAbs *via* the activation of complement and increased uptake by phagocytic cells and red blood cells (Schellekens, 2002). Although adalimumab is humanized mAbs, adalimumab can still cause immune response to produce anti-adalimumab antibodies in rats due to its immunogenicity for rats (Piccand et al., 2016). The drugs with immunosuppressive effect like methotrexate reduce the production of anti-adalimumab antibodies by suppressing the immune response (Ducourau et al., 2020). In this study, the production of anti-adalimumab antibodies was observed in rats with CIA under the continuous treatment of adalimumab, which was consistent with the results of other study (Piccand et al., 2016). More interestingly, a comparative level of anti-adalimumab antibodies was also observed between the rats in the adalimumab-treated and delanzomib + adalimumab-treated groups, suggesting that delanzomib less affected the expression of anti-adalimumab antibodies. Unlike the way by which methotrexate affected the pharmacokinetics of adalimumab, the formation of anti-adalimumab antibodies might not play a key role in the interaction between delanzomib and adalimumab in rats of CIA.

The mechanism by which delanzomib increased the level of FcRn in tissues was also investigated in this study. It might be the degradation of FcRn rather than the production of FcRn that was affected by delanzomib. The degradation of FcRn was found to be suppressed after the treatment of delanzomib. FcRn is a membrane protein mainly eliminated through the ubiquitin-proteasome pathway (Ma et al., 2018). Presumably, the degradation of FcRn was suppressed by delanzomib might derive from the inhibition of the proteasome activity by delanzomib. If true, other proteasome inhibitors such as bortezomib could also suppress the degradation of FcRn to affect the elimination of mAbs. Proteasome inhibitors including bortezomib have been used for the treatment of some systemic autoimmune diseases, and they may be simultaneously used with some anti-TNF-α mAbs in some cases (Verbrugge et al., 2015; Lassoued et al., 2019). The potential interactions between proteasome inhibitors and anti-TNF-α mAbs may affect the pharmacodynamics and pharmacokinetics of anti-TNF-α mAbs. In such case, the results of this work might have clinical relevance for patients who simultaneously take proteasome inhibitors and anti-TNF-α therapeutic proteins.

Although the treatment of delanzomib combined with adalimumab might be potentially beneficial for patients with RA, the safety of this potential therapy needs further evaluation, especially in the case that the dose of delanzomib is increased in the clinical treatment, because delanzomib has been reported to have strong nephrotoxicity similar to bortezomib (Ruggeri et al., 2009; Fan et al., 2021). Moreover, further research is also needed to clarify the mechanism by which delanzomib suppressed the degradation of FcRn.

## Conclusion

Taken together, the treatment of delanzomib combined with adalimumab may be a potential therapeutic approach for the treatment of RA by synergistically reducing the level of TNF-α *in vivo*. More importantly, it was initially confirmed that the PK interaction occurred between delanzomib and adalimumab to further prolong the anti-TNF-α effect of adalimumab, which was likely mediated by the effect of delanzomib on the levels of FcRn. This interesting finding might have clinical relevance for patients who simultaneously take proteasome inhibitors and anti-TNF-α therapeutic proteins.

## Data Availability

The original contributions presented in the study are included in the article/supplementary material, further inquiries can be directed to the corresponding authors.

## Abbreviations

ADAs, anti-drug antibodies; ANOVA, one-way analysis of variance; CIA, collagen-induced arthritis; CRP, C-reactive protein; DDI, drug-drug interaction; DL, delanzomib; ELISA, enzyme-linked immunosorbent assay; FcRn, neonatal Fc receptor; HRP, horseradish peroxidase; IL, interleukin; mAbs, monoclonal antibodies; PBS, phosphate-buffered saline; PCR, polymerase chain reaction; PK, pharmacokinetic; RA, rheumatoid arthritis; TMB, tetramethyl benzidine; TNF-α, tumor necrosis factor alpha.
